# *Hmga1/Hmga2* double knock-out mice display a “superpygmy” phenotype

**DOI:** 10.1242/bio.20146759

**Published:** 2014-04-11

**Authors:** Antonella Federico, Floriana Forzati, Francesco Esposito, Claudio Arra, Giuseppe Palma, Antonio Barbieri, Dario Palmieri, Monica Fedele, Giovanna Maria Pierantoni, Ivana De Martino, Alfredo Fusco

**Affiliations:** 1 Istituto di Endocrinologia ed Oncologia Sperimentale del CNR c/o Dipartimento di Medicina Molecolare e Biotecnologie Mediche, Facoltà di Medicina e Chirurgia di Napoli, Università degli Studi di Napoli “Federico II”, via Pansini 5, 80131 Naples, Italy; 2 Istituto Nazionale dei Tumori, Fondazione Pascale, 80131 Naples, Italy

**Keywords:** HMGA1, HMGA2, Pygmy, E2F

## Abstract

The *HMGA1* and *HMGA2* genes code for proteins belonging to the High Mobility Group A family. Several genes are negatively or positively regulated by both these proteins, but a number of genes are specifically regulated by only one of them. Indeed, knock-out of the *Hmga1* and *Hmga2* genes leads to different phenotypes: cardiac hypertrophy and type 2 diabetes in the former case, and a large reduction in body size and amount of fat tissue in the latter case. Therefore, to better elucidate the functions of the *Hmga* genes, we crossed *Hmga1*-null mice with mice null for *Hmga2*. The *Hmga1^−/−^/Hmga2^−/−^* mice showed reduced vitality and a very small size (75% smaller than the wild-type mice); they were even smaller than pygmy *Hmga2*-null mice. The drastic reduction in E2F1 activity, and consequently in the expression of the E2F-dependent genes involved in cell cycle regulation, likely accounts for some phenotypic features of the *Hmga1^−/−^/Hmga2^−/−^* mice.

## INTRODUCTION

The High Mobility Group A (HMGA) family comprises four proteins: HMGA1a, HMGA1b, HMGA1c and HMGA2. The *HMGA1* gene codes for the first three proteins by alternative splicings, whereas the HMGA2 protein is encoded by a distinct gene named *HMGA2* (Johnson et al., 1989). These proteins bind the minor groove of AT-rich DNA sequences through three short basic repeats, called “AT-hooks”, located at the NH_2_-terminal region of the proteins. HMGAs act exclusively as architectural proteins. Indeed, they have no transcriptional activity *per se*, but they are able to alter the chromatin structure by interacting with the transcription machinery, and thus they can negatively or positively regulate the transcriptional activity of several genes (Johnson et al., 1989; Reeves and Nissen, 1990; Thanos et al., 1993).

Both *HMGA* genes are widely expressed during embryogenesis, whereas their expression is absent or very low in adult tissues (Zhou et al., 1995; Chiappetta et al., 1996). In particular, *Hmga2* has not been detected in any of the adult mouse and human tissues tested, apart from a very low expression in CD34-positive hematopoietic stem cells, mouse preadipocytic proliferating cells and meiotic and post-meiotic cells (secondary spermatocytes and spermatids) (Chieffi et al., 2002). Conversely, *Hmga1* is expressed, albeit at low levels, in adult murine and human tissues. However, the expression of both *HMGA* genes becomes abundant in malignant cells *in vitro* and *in vivo* (Giancotti et al., 1989; Wood et al., 2000; Fedele et al., 2001; Fusco and Fedele, 2007). Indeed, HMGA proteins are highly expressed in all malignant tissues analyzed, namely, pancreas, thyroid, colon, breast, lung, ovary, uterine cervix, prostate and gastric carcinomas, squamous carcinomas of the oral cavity, and head and neck tumors (Fusco and Fedele, 2007). Moreover, HMGA protein expression is associated with a highly malignant phenotype as shown by the significant correlation detected between high levels of HMGA1 protein expression and both the presence of lymph-node metastasis and advanced clinical stage (Balcerczak et al., 2003; Flohr et al., 2003; Donato et al., 2004).

Several studies indicate that *HMGA* gene expression plays a causal role in carcinogenesis. Indeed, blockage of their expression prevents thyroid cell transformation (Berlingieri et al., 1995; Berlingieri et al., 2002) and leads to the death of malignant cells (Scala et al., 2000). Moreover, HMGA1 or HMGA2 overexpression is able to transform mouse and rat fibroblasts (Fedele et al., 1998; Fedele et al., 2001), and transgenic mice overexpressing either HMGA1 or HMGA2 develop haematopoietic malignancies and pituitary adenomas (Battista et al., 1999; Arlotta et al., 2000; Fedele et al., 2002; Fedele et al., 2005; Zaidi et al., 2006).

The high expression of HMGA proteins during embryogenesis suggests that they exert an important role in development. Indeed, the phenotypic characterization of mice knocked out (KO) for each of the *Hmga* genes revealed that these proteins play crucial roles in various aspects of development (Fusco and Fedele, 2007). Cardiac hypertrophy and type 2 diabetes were observed in *Hmga1*-null (A1-KO) and heterozygous mice suggesting that an appropriate amount of HMGA1 protein is required for cardiomyocytic cell growth and regulation of the insulin pathway (Foti et al., 2005; Fedele et al., 2006a). Conversely, *Hmga2*-null (A2-KO) and heterozygous mice showed a pygmy phenotype with a decrease in body size of 60% and 25%, respectively, compared with the wild-type (WT) mice, and a drastic reduction of fat tissue (Zhou et al., 1995; Anand and Chada, 2000), which suggests that HMGA2 plays an important role in the control of body size and adipocyte proliferation and differentiation.

However, since many genes are regulated by both *HMGA* proteins, it is obvious that several functions exerted by one member of the HMGA family are, at least partially, compensated by the other family member. Therefore, the aim of our work was to generate mice carrying an impairment of both *Hmga* genes. These mice were generated by crossing the *Hmga1*-null mice (A1-KO) (Fedele et al., 2006a) with mice null for *Hmga2* (A2-KO) (Zhou et al., 1995; Scala et al., 2001) and the resulting phenotype was analyzed. The *Hmga1/Hmga2*-null (A1/A2-KO) mice presented reduced vitality (most of them died in utero) and a very small size, even lower than that of the pygmy A2-KO mice. We demonstrate that E2F1 activity is impaired in A1/A2-KO mouse embryonic fibroblasts (MEFs) and tissues, an event that results in a drastic reduction of the expression of E2F1-dependent genes, which probably accounts for the “superpygmy” phenotype of the A1/A2-KO mice.

## MATERIALS AND METHODS

### Generation and genotyping of mutant mice

The A1/A2-KO mice were generated crossing A1-KO (Fedele et al., 2006a) and A2-KO (Zhou et al., 1995; Scala et al., 2001) null mice. The genotype of the A1-KO, A2-KO and A1/A2-KO mice was analyzed as previously described (Fedele et al., 2006a; Zhou et al., 1995).

A1-KO mice, originally generated in a mixed genetic background C57/Sv129, were backcrossed for more than 10 generations in C57Bl/6J. These mice can be estimated to derive more than 99% of their genes from the C57BL/6J strain. A2-KO is a spontaneous mutant pygmy mouse obtained from the Jackson Laboratory (Bar Harbor, ME). The original strain was C3H/HeNIcrWf. A1-KO and A2-KO were crossed to obtain double mutant mice. These mice were intercrossed for more than 20 generations. Thus, all the analyzed mice have the same genetic background.

All mice were maintained under standardized non-barrier conditions in the Laboratory Animal Facility of the Istituto dei Tumori di Napoli (Naples, Italy) and all studies were conducted in accordance with Italian regulations for experimentations on animals.

### Isolation of mRNA, Northern blot analysis and RT-PCR

Total RNA was extracted using TRI-reagent solution (Invitrogen, Carlsbad, CA) according to the manufacturer's protocol. cDNA was synthesized from total RNA using random hexamers (100 mM) and MuLV reverse transcriptase (Perkin Elmer, Santa Clara, CA). Semi-quantitative PCRs were run using the Gene Amp PCR System 9700 (Applied Biosystems, Foster City, CA). Specific PCR conditions are available upon request. RNA samples that had not been reverse transcribed before PCR served as negative control. For semiquantitative PCR, reactions were optimized for the number of cycles to ensure product intensity within the linear phase of amplification. The PCR products were separated on a 2% agarose gel, stained with ethidium bromide. Gels were scanned with Chemidoc (Bio-Rad, Hercules, CA). The primer sequences used to amplify the indicated mouse genes are available upon request.

### Growth curves of WT, A1-KO, A2-KO and A1/A2 KO MEFs

Primary MEFs were obtained from 12.5-day-old embryos. The MEFs were minced and used to establish single cell suspensions. They were grown in Dulbecco's modified Eagle's medium (DMEM) (GIBCO, Carlsbad, CA) containing 10% fetal bovine serum (Sigma, St Louis, MO), 1% glutamine (GIBCO), 1% penicillin/streptomycin and 1% gentamicin (GIBCO). Three MEF preparations for each genotype were plated in triplicate in a series of 6-cm culture dishes (4×10^4^/dish) and counted daily with a cell counter for 6 consecutive days to extrapolate growth curves. The values represent means ± SEM of the three MEF preparations for each genotype.

### Body weight, body length and growth analysis

The mice were generally kept and bred with ad libitum access to water and pelleted standard food (Mucedola, Milano, Italy). The body weight was measured monthly, beginning at 4 weeks of age, for 12 months. Body length was measured by manual immobilization, extension and measurement of the naso–anal length by a ruler.

### Senescence associated-β-galactosidase assay

4×10^4^ MEFs, plated 24 h before the assay, were washed twice with PBS and immersed in fixation buffer [2% (w/v) formaldehyde, 0.2% (w/v) glutaraldehyde in PBS] for 7 min. After three additional PBS washes, the cells were allowed to stain overnight in staining solution (40 mM citric acid/sodium phosphate pH 6.0, 150 mM NaCl, 2.0 mM MgCl_2_, 1 mg/ml X-gal) at 37°C without CO_2_ to avoid changes in pH. We used three MEF preparations for each genotype, plated in triplicate. Pictures of each preparation of MEFs were done at least three times for each replicate. The values represent the means ± SEM of three MEF preparations for each genotype. We represent percentage of MEFs stained for β-galactosidase activity versus the total of MEFs. Pictures were taken on a bright field microscope (Zeiss, Oberkochen, Germany) at a magnification of 50×.

### Protein extraction and Western blotting

Protein extraction and Western blotting procedures were carried out as reported elsewhere (Federico et al., 2009; Esposito et al., 2010). Briefly, for total cell extracts, cells were lysed in RIPA buffer (20 mM Tris-HCl pH 7.5, 5 mM EDTA, 150 mM NaCl, 1% Nonidet P40, and a mix of protease inhibitors), and clarified by centrifugation at 13,000 rpm at 4°C for 30 min. Total proteins were directly resolved on 12.5% polyacrylamide gel under denaturing condition and transferred to nitrocellulose filters for western blot analyses. Membranes were blocked with 5% BSA in TTBS and incubated with the specific primary antibodies. The antibodies used were: anti-β-actin (sc-1616, Santa Cruz Biotechnology, Inc., Santa Cruz, CA), anti-γ-tubulin (sc-17787, Santa Cruz), anti-vinculin (sc-7649, Santa Cruz), anti-p21 (sc-397, Santa Cruz), anti-p16Ink4a (ab-54210, Abcam, Cambridge, UK), anti p-19 (Ab102848, Abcam), anti p-27 (610241, BD Transduction Laboratories™), anti-cyclin E (sc-481, Santa Cruz), anti-cyclin A (sc-751, Santa Cruz). Antibodies versus the HMGA1 and HMGA2 proteins have been previously described (Pierantoni et al., 2003; Chiappetta et al., 2008). Membranes were then incubated with the horseradish peroxidase-conjugated secondary antibody (1:3000) for 60 min (at room temperature) and the reaction was detected with a western blotting detection system (ECL) (Promega, Fitchburg, WI).

### Statistical analyses

All values were tested for normal distribution using the D'Agostino–Pearson test for all variables. Since all values passed the test (P>0.05), parametric tests were used for the analysis. We used Chi squared to compare the Mendelian inheritance of A1/A2-KO mice. The association between body weight and body length in mice, MEF growth curves, senescence with the different genotypes were determined by one-way ANOVA followed by the Bonferroni post-test. The statistical analyses were performed using GraphPad Prism v.6.0 (La Jolla, CA, USA). A P value <0.05 was considered statistically significant.

## RESULTS

### Generation of A1/A2-KO mice

To generate A1/A2-KO mice, we crossed A1-KO mice with *Hmga2^+/−^* mice. The A2-KO mice in our animal house are infertile consequent to the block of spermatogenesis (Chieffi et al., 2002). We were able to produce mice with different combinations of *Hmga1* and *Hmga2* null alleles, including the A1/A2-KO mice. Mating A1^+/+^ A2^+/−^ × A1^+/+^ A2^+/−^ mice we generated 47 A1-KO/A2^+/+^ mice (37%), 64 A1-KO/A2^+/−^ mice (50%) and 17 A1/A2-KO mice (13%) of 128 total mice. Therefore, the generation of the double KO mice was much lower than expected according to Mendelian laws thereby indicating lethality of these embryos (P<0.05).

**Fig. 1.**
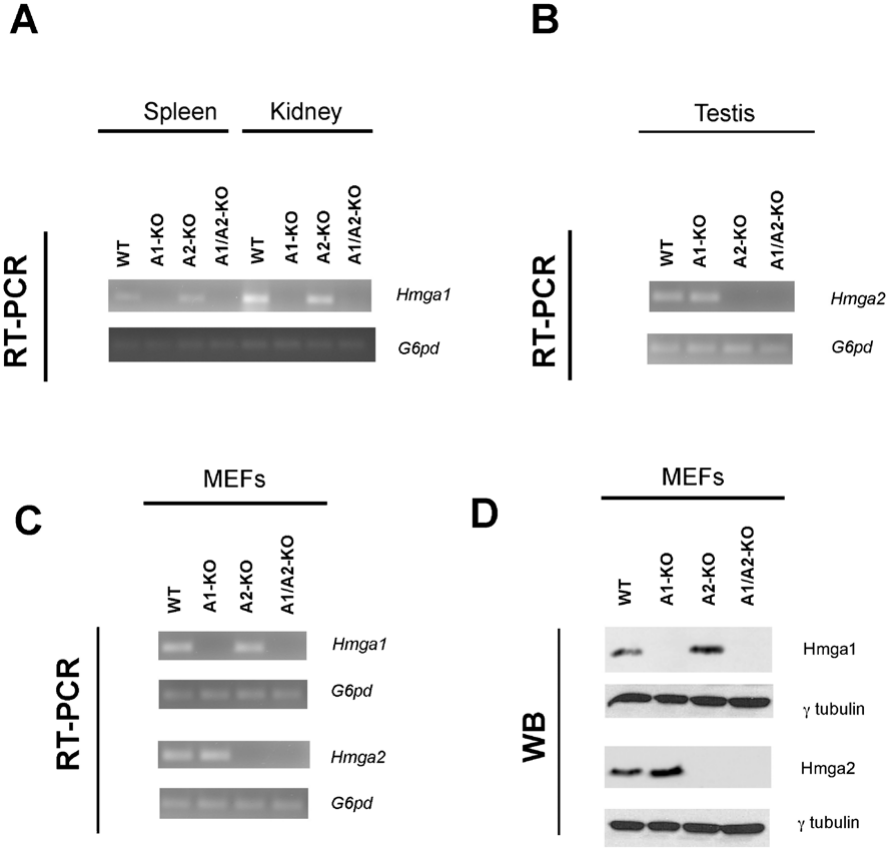
Lack of *Hmga1* and *Hmga2* expression in A1/A2-KO mice. (A) RT-PCR expression analysis of the *Hmga1* gene in WT, A1-KO, A2-KO and A1/A2-KO spleen and kidney tissue. (B) RT-PCR expression analysis of *Hmga2* gene in WT, A1-KO, A2-KO and A1/A2-KO testis tissue. (C) RT-PCR expression analysis of *Hmga1* and *Hmga2* genes in WT, A1-KO, A2-KO and A1/A2-KO MEFs at passage 4. G6PD gene expression was used as internal control. (D) Western blot analysis of Hmga1 and Hmga2 proteins in WT, A1-KO, A2-KO and A1/A2-KO protein extracted from MEFs at passage 4. γ-tubulin was used as loading control.

To verify the absence of *Hmga1* and *Hmga2* gene expression in the A1/A2-KO mice we performed RT-PCR and Western blot analyses. As shown in Fig. 1A, there was no *Hmga1* expression in kidney and spleen from the A1/A2-KO mice. Similarly, *Hmga2* expression was not detected in the testis (one of the few adult tissues expressing *Hmga2*) of A1/A2-KO mice, whereas it was present in the testis of WT and A1-KO animals (Fig. 1B). The same results were obtained from the analysis of the expression of *Hmga1* and *Hmga2* in MEFs from A1/A2-KO mice (Fig. 1C). Western blot analysis using antibodies raised against the Hmga1 and Hmga2 proteins did not reveal these proteins in the A1/A2-KO MEFs thereby confirming the RT-PCR data (Fig. 1D).

### The A1/A2-KO mice display a striking reduction of body size

**Fig. 2.**
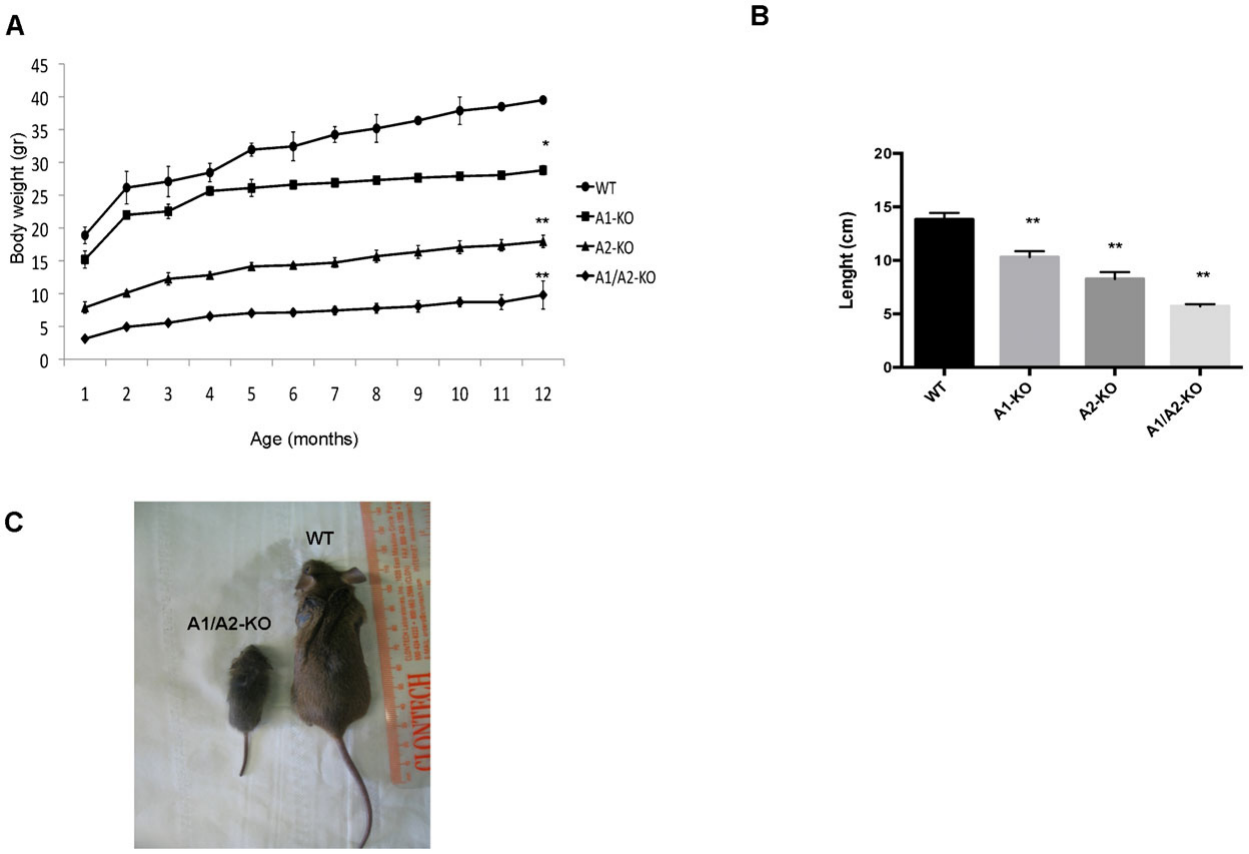
Body weight and size analysis of A1/A2-KO mice. (A,B) Representation of body weight, from 1 to 12 months, and naso–anal length, measured at 12 months of age, of cohorts, males or females, of 10 WT (circle), A1-KO (square), A2-KO (triangle) and A1/A2-KO (diamond) mice. Values are means ± SEM. *P<0.05, **P<0.01 versus WT. (C) Gross appearance of a representative 6-month-old A1/A2-KO mouse and a sex-matched WT sibling.

At birth, the A1/A2-KO mice were smaller than the WT, A1-KO and even the A2-KO littermates, which have been described as “pygmy mice” (Zhou et al., 1995). The differences in body size among these mice remained evident in adulthood (Fig. 2). Therefore, we monitored the weight and growth-length of the WT, A1-KO, A2-KO and A1/A2-KO mice. The A1/A2-KO mice weighed much less than each single KO mice, even one month after birth. At 12 months of age, body weight was reduced by 27%, 55% and 75% in the A1-KO, A2-KO and A1/A2-KO mice, respectively, compared with WT (*P<0.05, **P<0.01) (Fig. 2A). Also body length (nose-to-tail), at 12 months was markedly reduced: A1-KO mice were 25%, A2-KO 40% and A1/A2-KO 60% shorter than WT mice (**P<0.01) (Fig. 2B). Interestingly, the size and the weight of livers and spleens of A1/A2-KO mice are about one forth of the WT organs (data not shown). These results suggest that the *Hmga1* and *Hmga2* genes play a critical role in controlling body growth. A representative A1/A2-KO mouse is shown in Fig. 2C. We decided to call the A1/A2-KO mice “superpygmy” to distinguish them from the A2-KO pigmy mice.

### A1/A2-KO MEFs have a lower growth rate than WT, A1-KO and A2-KO MEFs

**Fig. 3.**
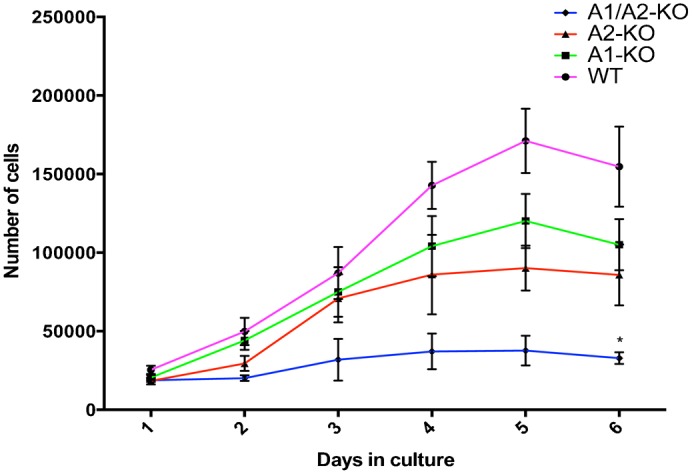
Representative growth curve of WT, A1-KO, A2-KO and A1/A2-KO MEFs. MEFs from WT (circle), A1-KO (square), A2-KO (triangle) and A1/A2-KO (diamond) embryos at 12.5 dpc, at culture passage 4, were plated and counted daily for 6 days to extrapolate growth curves. The growth curves refer to three MEF preparations for each genotype plated in triplicate. Values represent means ± SEM. *P<0.05 versus WT.

**Fig. 4.**
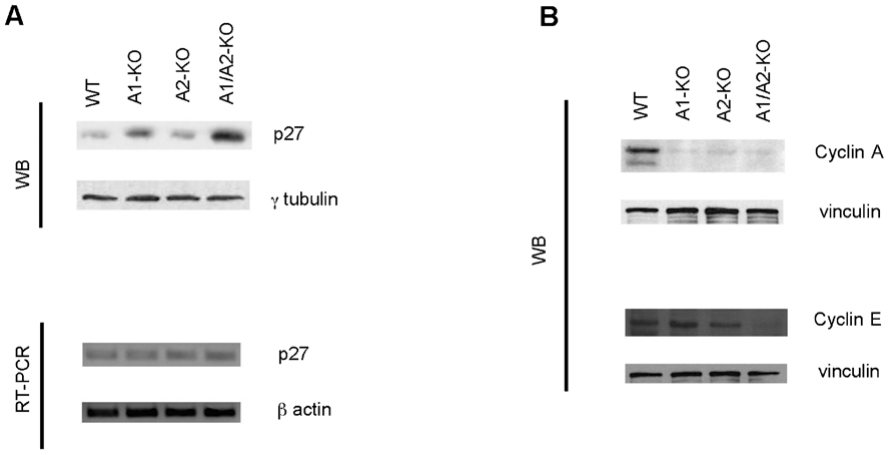
Expression of p27, cyclin A and cyclin E in A1/A2-KO MEFs. (A) Expression of cell cycle inhibitor p27 in representative MEFs from each genotype was determined by RT-PCR and Western blot at culture passage 4. γ-tubulin was used as loading control and β-actin gene expression was used as internal control. (B) Expression of cyclin A and cyclin E in representative MEFs from each genotype was determined by Western blot at culture passage 4. Vinculin was used as loading control.

We analyzed the growth rate of the MEFs derived from WT, A1-KO, A2-KO and A1/A2-KO mice, established from embryos at 12.5 dpc, by performing a growth curve. As shown in Fig. 3, growth rate was much lower in A1/A2-KO MEFs than in WT MEFs (*P<0.05). The analysis of the genes coding for proteins involved in cell cycle control revealed higher p27 protein levels in the A1/A2-KO MEFs than in the WT (Fig. 4A). Since we found no differences at mRNA level (Fig. 4A), it is likely that post-transcriptional mechanisms are involved in the control of this protein in A1/A2-KO MEFs. Conversely, cyclin A and cyclin E expression was decreased in all *Hmga*-null MEFs particularly in those deriving from the A1/A2-KO mice (Fig. 4B).

**Fig. 5.**
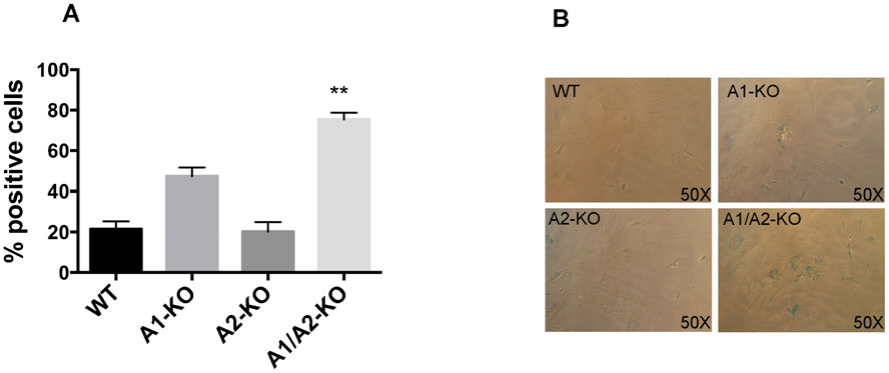
Senescence assay of MEFs from WT, A1-KO, A2-KO and A1/A2-KO mice. (A) Percentage of MEFs stained for β-galactosidase activity versus the total of MEFs. The values represent the means ± SEM of three MEF preparations for each genotype. (B) Light microscopy of representative WT, A1-KO, A2-KO and A1/A2-KO MEFs stained for β-galactosidase activity at culture passage 4. Pictures were taken on a bright field microscope (Zeiss) at a magnification of 50×. **P<0.01 versus WT.

Next we examined the susceptibility to senescence of the *Hmga*-null MEFs at different culture passages by measuring senescence-associated β-galactosidase (SA-β-gal) activity. As shown in Fig. 5A at passage 4, the number of cells showing SA-β-gal activity was significantly higher in A1/A2-KO MEFs than in WT (**P<0.01 versus WT). A representative picture of the senescence assay is shown in Fig. 5B.

**Fig. 6.**
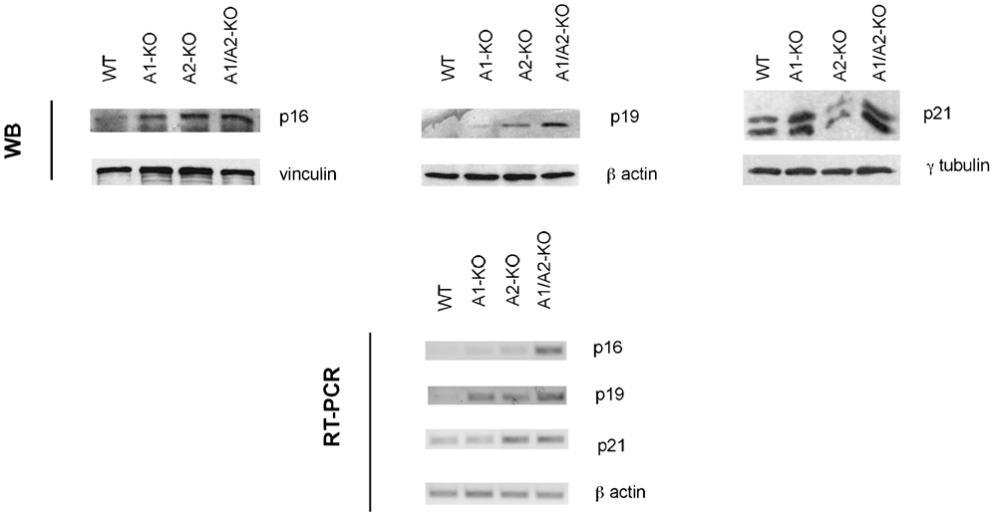
Analysis of p16, p19 and p21 expression in WT, A1-KO, A2-KO and A1/A2-KO MEFs. Expression of cell cycle inhibitors p16, p19 and p21 in representative MEFs from each genotype was determined by Western blot and RT-PCR at culture passage 7. β-actin gene expression was used as internal control. Vinculin, β-actin and γ-tubulin were used as loading control.

Senescent MEFs express elevated levels of p16, p19 and p21 consequent to replication and culture stress (Narita et al., 2006). These genes may cooperate to inhibit Rb phosphorylation and maintain growth arrest in an irreversible state. Therefore, we evaluated p16, p19 and p21 mRNA and protein levels in WT, A1-KO, A2-KO and A1/A2-KO MEFs by quantitative RT-PCR and Western blot analysis. As shown in Fig. 6, the mRNA and protein expression levels of p16, p19 and p21 at passage 7 were significantly higher in A1/A2-KO than in WT MEFs.

### Reduced E2F1 activity may account for the “superpygmy” phenotype

We previously demonstrated that HMGA1 and HMGA2 overexpression enhances E2F1 activity by binding to RB and displacing HDAC from the RB/E2F complex (Seville et al., 2005; Fedele et al., 2006b). Therefore, we envisaged that the “super-pygmy” phenotype of A1/A2-KO mice could result from decreased E2F1 activity that would, in turn, lead to reduced cell growth and, maybe, embryonic stem cell self-renewal.

**Fig. 7.**
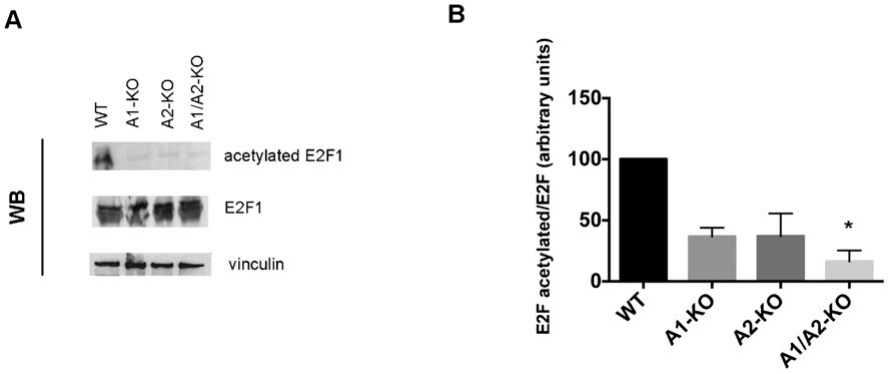
E2F1 acetylation in A1/A2-KO MEFs. (A) Western blot analysis of acetylated and total form of E2F1 in representative WT, A1-KO, A2-KO and A1/A2-KO MEFs. Vinculin was used as loading control. (B) Densitometric analysis of the levels of acetylated E2F1 versus E2F1 total in WT, A1-KO, A2-KO and A1/A2-KO MEFs. Level of acetylated E2F1 versus E2F1 total in WT was assumed as 100 (arbitrary units). These values represent the means ± SEM of two experiments. *P<0.05 versus WT.

Since E2F1 acetylation reflects its activation augmenting DNA binding and stabilizing the protein (Martínez-Balbás et al., 2000) thereby enhancing its transcriptional activity, we evaluated the amount of acetylated E2F1 in WT, A1-KO, A2-KO and A1/A2-KO MEFs by western blot analysis using antibodies specific for the acetylated form of E2F1. A representative experiment is shown in Fig. 7A. Densitometric analysis deriving from two Western blot experiments showed that the levels of acetylated E2F1 were about 60% lower in the A1-KO and A2-KO MEFs than in the WT MEFs (Fig. 7B). A further decrease was observed in the A1/A2-KO MEFs, where it is almost undetectable. Consistently, the expression of cyclin A (Fig. 4B) and cyclin E (Fig. 4B), which are specific targets of E2F1, paralleled the levels of acetylated E2F (Fig. 7).

## DISCUSSION

Because proteins HMGA1 and HMGA2 proteins share the ability to regulate a number of common genes, some functions might be compensated by the expression of the other member of the HMGA family. Therefore, to identify all the activities of the HMGA proteins we generated mice KO for both the genes by crossing the A1-KO mice with A2-KO mice. Only a few A1/A2-KO animals were generated, which is indicative of embryonic lethality. Moreover, most of them died before one year after the birth. Therefore, we postulate that the HMGA proteins play a critical role in controlling some functions that are essential for embryonic and/or fetal development. It is noteworthy that the presence of the other family member was sufficient to prevent embryo lethality in the A1-KO and A2-KO mice. Preliminary results from our laboratory indicate that most of the deaths were due to haemorrhagic events that may result from increased levels of angiogenic factors (A. Federico and F.E., unpublished data).

The most impressive feature of the A1/A2-KO mice is their size: they are about 75% smaller than the WT littermates. We called these mice “superpygmy” since they are even much smaller than the pygmy A2-KO mice. This result is not surprising since the *Hmga2* gene is involved in defining the body size in mice and, even in humans. Indeed, mice overexpressing *Hmga2* showed a giant phenotype that was a mirror image of the pygmy phenotype (Battista et al., 1998; Battista et al., 1999; Baldassarre et al., 2001). Moreover, *HMGA2* is an important genetic determinant for human adult height (Weedon et al., 2008; Buysse et al., 2009; Hodge et al., 2009; Yang et al., 2010; Hendriks et al., 2011; Carty et al., 2012; Makvandi-Nejad et al., 2012). Consistently, an 8-year-old boy carrying a truncated *HMGA2* gene displayed extreme somatic overgrowth with features strikingly similar to those observed in the *Hmga2* transgenic mice (Ligon et al., 2005). However, our results implicate also *Hmga1* in body size determination. In fact, a slight reduction in size occurred also in the A1-KO mice, while impairment of both *Hmga* genes leads to a drastic reduction in body size. Moreover, we found a reduced body size already at birth; in fact, A1/A2-KO MEFs grew slower than both the WT MEFs and those carrying the impairment of a single *Hmga* gene.

We also found that E2F1 activity was impaired in the absence of HMGA expression, as shown by its very low acetylation status. Impaired E2F1 activity may account for the “superpygmy” phenotype of the A1/A2-KO mice and the slower growth of the corresponding MEFs. Indeed, E2F1 activity was very low in A1/A2-KO MEFs. This result is consistent with our previous finding that HMGA2 displaces histone deacetylase 1 from the pRB/E2F1 complex thereby resulting in enhanced acetylation of both E2F1 and DNA-associated histones, and thus promoting E2F1 activation. Accordingly, the level of the acetylated form of E2F1 was drastically lower in all the *Hmga* KO mice, particularly in the A1/A2-KO mice, than in the WT. This is consistent with the reduced expression of the E2F-dependent genes, namely, cyclin A and cyclin E, in the A1/A2-KO MEFs. However, it cannot be excluded that other genes regulated by the HMGA genes might contribute to the phenotype of the A1/A2-KO mice.

In contrast with Narita et al., who reported that *HMGA1* reduces cell lifespan (Narita et al., 2006), A1-KO and A1/A2-KO MEFs became senescent earlier than WT MEFs. This result suggests that the cellular context plays a critical role in determining the effect of the HMGA proteins on cell growth. In the case of A1-KO MEFs, the context may be considered more physiological than experiments in which *HMGA1* was ectopically overexpressed in IMR90 (Narita et al., 2006). It is likely that *HMGA* overexpression causes drastic changes in the chromatin architecture that causes non-cancerous cells to become senescent. Moreover, the different experimental approach, one *in vivo* and the previous one *in vitro*, may account for these contradictory results. Indeed, significant discrepancies were found between *in vitro* and transfection approaches in a study of the p53 pathway (Toledo and Wahl, 2006). Noteworthy, the behaviour of the *Hmga*-null MEFs in our study is in agreement with the notion that HMGA overexpression plays an oncogenic role, which is a feature of malignant neoplasias.

In conclusion, the generation of mice carrying the impairment of both the *Hmga* genes is an excellent model with which to shed light on the functions of these genes.
